# High-Efficiency Super-Resolution FMCW Radar Algorithm Based on FFT Estimation

**DOI:** 10.3390/s21124018

**Published:** 2021-06-10

**Authors:** Bong-seok Kim, Youngseok Jin, Jonghun Lee, Sangdong Kim

**Affiliations:** 1 Division of Automotive Technology, Daegu Gyeongbuk Institute of Science and Technology (DGIST), Daegu 42988, Korea; remnant@dgist.ac.kr (B.-s.K.); ysjin@dgist.ac.kr (Y.J.); jhlee@dgist.ac.kr (J.L.); 2 Department of Interdisciplinary Engineering, DGIST, Daegu 42988, Korea

**Keywords:** FMCW radar, super-resolution, low complexity, MUSIC

## Abstract

This paper proposes a high-efficiency super-resolution frequency-modulated continuous-wave (FMCW) radar algorithm based on estimation by fast Fourier transform (FFT). In FMCW radar systems, the maximum number of samples is generally determined by the maximum detectable distance. However, targets are often closer than the maximum detectable distance. In this case, even if the number of samples is reduced, the ranges of targets can be estimated without degrading the performance. Based on this property, the proposed algorithm adaptively selects the number of samples used as input to the super-resolution algorithm depends on the coarsely estimated ranges of targets using the FFT. The proposed algorithm employs the reduced samples by the estimated distance by FFT as input to the super resolution algorithm instead of the maximum number of samples set by the maximum detectable distance. By doing so, the proposed algorithm achieves the similar performance of the conventional multiple signal classification algorithm (MUSIC), which is a representative of the super resolution algorithms while the performance does not degrade. Simulation results demonstrate the feasibility and performance improvement provided by the proposed algorithm; that is, the proposed algorithm achieves average complexity reduction of 88% compared to the conventional MUSIC algorithm while achieving its similar performance. Moreover, the improvement provided by the proposed algorithm was verified in practical conditions, as evidenced by our experimental results.

## 1. Introduction

Radar sensors are a subject of research in various fields, such as defense, space, and vehicles, given their robustness against several conditions, including wind, rain, fog, light, humidity, and temperature [1,2,3,4,5,6]. The ultra wide band (UWB) radar systems with high-resolution and high-precision had been in the spotlight as a representative radar system [3]. The UWB radar systems employ very narrow pulse width and thus they require very wide bandwidth [7,8]. This is the reason for the high complexity of the UWB radar systems. Hence, UWB radar systems are mainly used in fields that are less sensitive to the burden of costs such as defense and space [9]. As the application range of radar gradually expands, those with low cost and low complexity have been placed in the research spotlight among the many types of radar systems. The continuous wave (CW) radar is a representative low-complexity radar system [10,11]. The CW radar systems use only the difference between the carrier frequencies at the transmitter and receiver to estimate the velocity of the target. Since it is only necessary to perform sampling on the sine wave signal corresponding to the carrier frequency difference, it is converted into a digital signal with less complexity burden. However, the CW radar has the limitation that it cannot be used for various purposes because it cannot measure the distance to the target.

As an alternative to these, studies on frequency modulation continuous wave (FMCW) radar systems have been reported [12,13,14,15,16,17,18]. FMCW radar systems are capable of estimating the range, Doppler, and angle of targets, despite their low-cost, low-complexity hardware systems, as their signal processing is performed in a low frequency band after mixing. As the applications of the radar sensors increase, FMCW radar technology is considered one of the most promising technologies. For example, the FMCW radar systems have been applied to surveillance applications [19,20,21]. In [19,20], they have presented the design and test of radar sensing platform based on FMCW radar in transport systems. In addition, they provided for the better trade-off that can be found in terms of power consumption and the detectable range. In [21], however, the authors proposed a solution to rapidly detect the moving targets by utilizing the subtract between two FMCW chirp signals. In [22,23,24,25], they addressed that the FMCW radar is one of the most promising techniques for non-contact monitoring to measure vital signals, such as heart respiration rates. In [23], they showed the human indication by measuring the respiration pattern using the FMCW radar and deep learning algorithm. In [25], the authors presented a vital sign monitoring systems by using the 120 GHz FMCW radar. In [26], a low complexity FMCW radar algorithm was proposed by reducing the dimension of 2D data. Meanwhile, FMCW radar systems had been utilized for vehicles [27,28,29]. In [27], the randomized switched antenna arrays FMCW radar were introduced for automotive applications. They tried to solve the delay-space coupling problem of the traditional switched antenna arrays systems. In [28], they proposed a method to simultaneously detect and classify objects by using a deep learning model, specifically, that you only look once, so-called YOLO, with pre-processed automotive radar signals using FMCW radar.

In FMCW radar systems, meanwhile, fast Fourier transform (FFT)-based estimators are widely employed [19]. In [16], a novel direction of arrival (DOA) estimation algorithm was proposed for FMCW radar systems. This algorithm virtually extends the number of arrays using simple multiplications. The FFT is employed in this algorithm, and thus, the computational complexity of this algorithm is very low compared with other high-resolution algorithms. However, it does not provide a large resolution improvement, although the resolution provided by this algorithm is higher than that of conventional FFT-based estimation algorithms. In [18], an algorithm employing only regions of interest in the total samples was proposed, in order to estimate the distance and the velocity of targets in an attempt to reduce redundant complexity. However, an improvement in resolution was not expected, as this algorithm was also based on the FFT. In other words, it is difficult for FFT-based estimators to distinguish between multiple adjacent targets.

To overcome this disadvantage of FFT-based estimators in FMCW radar systems, several algorithms have been proposed. In [30,31,32,33,34,35,36,37,38,39], various super-resolution algorithms have been proposed, such as the multiple signal classification (MUSIC) and estimation of signal parameters via rotational invariance technique (ESPRIT) algorithms. These algorithms employ eigenvalue decomposition (EVD) or singular-value decomposition (SVD) of the correlation matrix obtained from the received signal, in order to distinguish signal and noise subspaces. The parameters corresponding to the desired signals are accurately estimated using the relationship that the subspace of the signal and the subspace of the noise are orthogonal to each other. However, their computational complexity drastically increases as the number of input samples increases. Thus, these algorithms may not be suitable when the number of input samples is large. Oh et al. [35] employed the inverse of the covariance matrix, instead of the EVD or SVD, to reduce the complexity of super-resolution algorithms. However, under a low signal-to-noise ratio (SNR), the performance of this algorithm was significantly degraded [36]. In [37], a low-complexity MUSIC algorithm for DOA estimation was proposed. This algorithm properly uses the trade-off between the field-of-view (FOV) and the angular resolution. Hence, this algorithm attempts to reduce the computational complexity of the MUSIC algorithm. In this case, however, DOA estimation is considered; thus, the number of inputs to the MUSIC algorithm to be considered is not large, as the maximum number of samples is the same as the number of arrays.

To reduce the computational complexity while exploiting the high-resolution features of super-resolution-based estimators, the algorithm proposed in this paper reduces the number of samples used as input to the MUSIC algorithm, based on the beat frequency estimated by the FFT. In other words, in the proposed algorithm, the number of samples is set based on the distance estimated by FFT, instead of the maximum detectable distance (as in the conventional MUSIC algorithm). Based on this reduced number of samples, the overall complexity of the proposed algorithm is decreased, by using only some of the samples of a given beat signal as the input to the MUSIC algorithm. Compared to [37], in the proposed algorithm, the number of samples used as inputs of the MUSIC algorithm is also determined in various ways according to various situations depending on the estimated distance rather than one threshold condition. To this end, in this paper, we mathematically show the process of how many reduced samples were required for the same performance according to the ratio of the distance estimated by the FFT and the maximum detection distance. Our simulation results confirm the improvement in performance produced by the proposed algorithm; that is, the proposed algorithm can achieve similar performance to the conventional MUSIC algorithm, despite its considerably lower complexity. Moreover, our experimental results verify that the proposed algorithm can operate well in a real environment.

The remainder of this paper is structured as follows: in Section 2, we describe the system model considered in this study. Then, the proposed low-complexity MUSIC algorithm is described in Section 3. In Section 4, through simulations, the performance of the proposed algorithm is compared to that of the conventional MUSIC algorithm and their computational complexities are evaluated. In Section 5, the experimental setup is introduced and the experiment results are provided, which confirm the performance of the proposed algorithm in practical environments. Finally, we conclude this paper in Section 6.

## 2. System Model for FMCW Radar Systems

The system model of a FMCW radar system consisting of one transmitting (TX) antenna and one receiving (RX) antenna is considered in this section.

**Figure 1:**
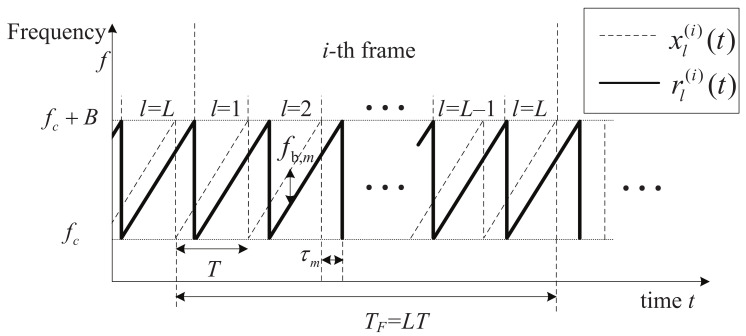
System model of an FMCW radar.

The TX signal at the ith frame of the FMCW radar is denoted by $x^{\left(i\right)}\left(t\right)$, which is transmitted from the TX antenna during $N_{F}$ frames, as shown in Figure 1.

The TX FMCW signal, $x^{\left(i\right)}\left(t\right)$, is composed of a total of *L* ramp signals, and is expressed as [17,18]

(1) $$\begin{array}{c} x^{\left(i\right)}\left(t\right)=\sum_{l=0}^{L-1}x_{0}\left(t-lT-iLT\right),\;\;\mathrm{for}\;\;0\leq i\leq N_{F}-1, \end{array}$$

where $x_{0}\left(t\right)$ is a ramp signal, expressed as

(2) $$\begin{array}{c} x_{0}\left(t\right)=\exp\left(j2\pi \left(f_{c}t+\frac{\mu }{2}t^{2}\right)\right),\;\;\mathrm{for}\;\;\;0\leq t\leq T, \end{array}$$

where $f_{c}$ is the center frequency and μ is the sweep rate of the ramp signal. Let *B* and *T* denote the system bandwidth and the time duration of the ramp signal $x_{0}\left(t\right)$, respectively. Hence, μ is calculated as μ=B/T. The TX signal $x^{\left(i\right)}\left(t\right)$ is reflected on *M* targets and is then received by the RX antenna. Let $r_{l}^{\left(i\right)}\left(t\right)$ denote the RX signal corresponding to the lth ramp signal, expressed as follows:

(3) $$\begin{array}{c} r_{l}^{\left(i\right)}\left(t\right)=\sum_{m=1}^{M}{\tilde{a}}_{m,l}^{\left(i\right)}x^{\left(i\right)}\left(t-\tau_{m}^{\left(i\right)}\right)\exp\left(j2\pi f_{D,m}^{\left(i\right)}\left(lT+iT_{F}\right)\right)+{\tilde{z}}_{l}^{\left(i\right)}\left(t\right)\\ \;\;\mathrm{for}\;\;0\leq l\leq L-1\;\;\mathrm{and}\;\;0\leq i\leq N_{F}-1, \end{array}$$

where ${\tilde{a}}_{m,l}^{\left(i\right)}$ is the complex amplitude component corresponding to the mth target, $f_{D,m}^{\left(i\right)}$ is the Doppler frequency due to the movement of the mth target, $\tau_{m}^{\left(i\right)}$ is the time delay due to the distance between the radar and the mth target, and ${\tilde{z}}_{l}^{\left(i\right)}$ is the complex additive white Gaussian noise (AWGN) component. By multiplying the conjugate of $x_{0}\left(t\right)$ (i.e., $x_{0}^{*}\left(t\right)$) by $r_{l}^{\left(i\right)}\left(t\right)$, the beat signal $y_{l}^{\left(i\right)}\left(t\right)$ is obtained and expressed as [37]

(4) $$\begin{array}{ccc} y_{l}^{\left(i\right)}\left(t\right) & = & x_{0}^{*}\left(t\right)\times r_{l}^{\left(i\right)}\left(t\right)\\ & = & \sum_{m=1}^{M}{\tilde{a}}_{m,l}^{\left(i\right)}\underset{\mathrm{Doppler}\;\mathrm{term},\;≜v_{m}^{(i)l}}{\underbrace{\exp\left(j2\pi f_{D,m}^{\left(i\right)}\left(lT+iT_{F}\right)\right)}}\underset{\;\mathrm{range}\;\mathrm{term},≜\eta_{m}^{\left(i\right)}\left(t\right)}{\underbrace{\exp\left(-j2\pi \mu \tau_{m}t\right)}}\\ & + & \underset{\mathrm{noise}\;\mathrm{term},\;≜z_{l}^{\left(i\right)}\left(t\right)}{\underbrace{{\tilde{w}}_{l}^{\left(i\right)}\left(t\right)x_{0}^{*}\left(t\right)}}. \end{array}$$

After analog/digital conversion (ADC), $y_{l}^{\left(i\right)}\left(t\right)$ changes into the sampled beat signal $y_{l}^{\left(i\right)}\left[n\right]$ with a sampling time interval $t_{s}$, expressed as

(5) $$\begin{array}{ccc} y_{l}^{\left(i\right)}\left[n\right] & = & \sum_{m=1}^{M}{\tilde{a}}_{m,l}^{\left(i\right)}\exp\left(j2\pi f_{D,m}^{\left(i\right)}\left(lT+iT_{F}\right)\right)\exp\left(-j2\pi \mu \tau_{m}^{\left(i\right)}nt_{s}\right)+z_{l}^{\left(i\right)}\left[n\right]\\ & = & \sum_{m=1}^{M}{\tilde{a}}_{m,l}^{\left(i\right)}\exp\left(j2\pi f_{D,m}^{\left(i\right)}\left(lT+iT_{F}\right)\right)\exp\left(-j2\pi f_{b,m}^{\left(i\right)}nt_{s}\right)+z_{l}^{\left(i\right)}\left[n\right]\\ & = & \sum_{m=1}^{M}{\tilde{a}}_{m,l}^{\left(i\right)}v_{m}^{(i)l}\eta_{m}^{\left(i\right)}\left[n\right]+z_{l}^{\left(i\right)}\left[n\right], \end{array}$$

where $f_{b,m}$ is the beat frequency (i.e., $f_{b,m}=\mu \tau_{m}^{\left(i\right)}$). Here, the number of total samples is denoted by $N_{s}$, and is expressed as

(6) $$\begin{array}{c} N_{s}=\left\lfloor T\cdot f_{s}\right\rfloor , \end{array}$$

where $f_{s}$ is the sampling frequency (i.e., $f_{s}=1/t_{s}$) and ⌊·⌋ is the floor operator. From Equation (6), it can be shown that the number of total samples $N_{s}$ is determined by the sampling frequency $f_{s}$ and *T*. In addition, $f_{s}$ is determined by the maximum detectable distance $d_{\max}$. Using the relation between $f_{s}$ and $d_{\max}$ [40,41], the minimum sampling frequency $f_{s,\min}$ is as follows:

(7) $$\begin{array}{c} f_{s,\min}=\frac{4\mu d_{\max}}{c}\leq f_{s}, \end{array}$$

where *c* is the speed of the electromagnetic wave. Hence, the minimum number of samples is denoted by $N_{s,\min}$, expressed as

(8) $$\begin{array}{ccc} N_{s,\min} & = & \left\lfloor \frac{4\mu d_{\max}}{Tc}\right\rfloor \\ & = & \left\lfloor \frac{4Bd_{\max}}{c}\right\rfloor . \end{array}$$

For simplicity, the sampled beat signals are considered for only one frame; thus, the frame index *i* is omitted. For example, $f_{b,m}^{\left(i\right)}$, $\tau_{m}^{\left(i\right)}$, and $\eta_{m}^{\left(i\right)}$ are changed to $f_{b,m}$, $\tau_{m}$, and $\eta_{m}$, respectively. By redefining the coefficient term $a_{m,l}$ (i.e., $a_{m,l}={\tilde{a}}_{m,l}^{\left(i\right)}v_{m}^{(i)l}$), the sampled beat signal can be simply expressed as [37]

(9) $$\begin{array}{c} y_{l}\left[n\right]=\sum_{m=1}^{M}a_{m,l}\eta_{m}\left[n\right]+z_{l}\left[n\right]. \end{array}$$

To effectively denote the variables, the variable is expressed, in vector form, as [37]

(10) $$\begin{array}{c} y_{l}=Ha_{l}+w, \end{array}$$

where $y_{l}\in C^{N_{s}\times 1}$ is a vector form of $y_{l}\left[n\right]$ (i.e., $y_{l}={\left[y_{l}\left[0\right],y_{l}\left[1\right],y_{l}\left[2\right],...,y_{l}\left[N_{s}-1\right]\right]}^{T}$), where ${\left(\cdot \right)}^{T}$ is the transpose operator, and $H\in C^{N_{s}\times M}$ and $a_{l}\in C^{M\times 1}$ are the matrix and vector corresponding to the beat signal η[n] and amplitude $a_{m,l}$, respectively, where $C^{N\times L}$ and $R^{N\times L}$ are denoted by N×L complex and real matrices, respectively, and $w\in C^{N_{s}\times 1}$ is an AWGN vector. The beat signal matrix, H, is composed of *M* beat signal column vectors $h_{m}\in C^{N_{s}\times 1}={\left[\eta_{m}\left[0\right],\eta_{m}\left[1\right],...,\eta_{m}\left[N_{s}-1\right]\right]}^{T}$ for 1≤m≤M, and is expressed as

(11) $$\begin{array}{ccc} H & = & \left[h_{1},h_{2},...,h_{M}\right]\\ & = & \left[\begin{array}{cccc} \eta_{1}\left[0\right] & \eta_{2}\left[0\right] & \cdots & \eta_{M}\left[0\right]\\ \eta_{1}\left[1\right] & \eta_{2}\left[1\right] & \cdots & \eta_{M}\left[1\right]\\ \vdots & \vdots & \ddots & \vdots \\ \eta_{1}\left[N_{s}-1\right] & \eta_{2}\left[N_{s}-1\right] & \cdots & \eta_{M}\left[N_{s}-1\right] \end{array}\right]. \end{array}$$

The lth amplitude vector $a_{l}$ and amplitude matrix A are expressed as

(12) $$\begin{array}{c} a_{l}={\left[a_{1,l},a_{2,l},...,a_{M,l}\right]}^{T}, \end{array}$$

(13) $$\begin{array}{c} A=\left[a_{1},a_{2},...,a_{L}\right]. \end{array}$$

As the range of the target is estimated based on the time delay $\tau_{m}$, the range of the target can be obtained in the FMCW radar system by estimating the beat frequency of $\eta_{m}\left[n\right]$, which is denoted by $f_{b,m}$. Let ${\hat{d}}_{m}$ denote the estimated range, which is calculated using the time delay as follows:

(14) $$\begin{array}{c} {\hat{d}}_{m}=\frac{\tau_{m}}{2}. \end{array}$$

By substituting $\tau_{m}=f_{b,m}/\mu$ into Equation (14), ${\hat{d}}_{m}$ can be obtained by estimating $f_{b,m}$, such that:

(15) $$\begin{array}{c} {\hat{d}}_{m}=\frac{f_{b,m}}{2\mu }. \end{array}$$

The range resolution, $d_{\Delta }$, is inversely proportional to the TX waveform bandwidth, *B*, as follows [42]:

(16) $$\begin{array}{c} d_{\Delta }=\frac{c}{2B}. \end{array}$$

By substituting Equation (8) into Equation (16), the range resolution, $d_{\Delta }$, can be expressed as

(17) $$\begin{array}{ccc} d_{\Delta } & = & \frac{2d_{\max}}{N_{s}}\\ & = & \frac{2d_{\max}}{Tf_{s}}. \end{array}$$

In pulsed radar systems, which is one of the representative radar systems, since bandwidth is a factor that determines the range resolution, increasing *T* or $N_{s}$ could not improve the range resolution. From Equation (17), however, in order to improve the resolution performance in FMCW radar systems, an increase in the duration of the FMCW TX signal *T* or an increase in the number of samples $N_{s}$ is required for a given $f_{s}$ (i.e., to decrease $d_{\Delta }$). [40,41] This implies that the computational complexity inevitably increases when improving the resolution performance.

## 3. Proposed Super-Resolution Algorithm Using FFT-Estimated Ranges

**Figure 2:**
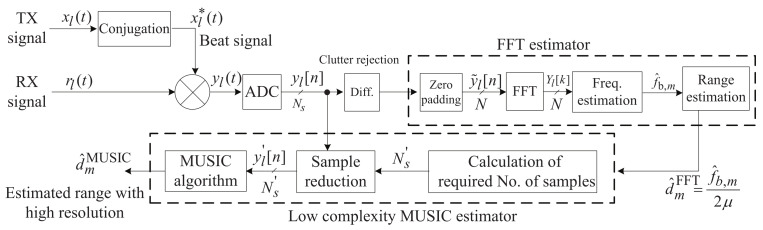
Block diagram of the proposed super-resolution algorithm.

This section describes the proposed low-complexity super-resolution FMCW radar algorithm. Figure 2 shows a block diagram of the proposed algorithm. The proposed algorithm consists of two steps: first, simple clutter is rejected and the ranges are coarsely estimated using the FFT; second, the computational complexity of the super-resolution algorithm is reduced using the estimation results from the first step, thus reducing the considered number of samples used as input to the super-resolution algorithm. For this study, we employed the MUSIC algorithm, which is a representative super-resolution algorithm, for comparative purposes. The details of each step are provided in each subsection.

### 3.1. Simple Clutter Rejection and Coarse Range Estimation Using FFT

As mentioned above, the proposed algorithm performs simple clutter rejection and coarse range estimation using the FFT. To achieve simple clutter rejection, the proposed algorithm determines the difference between $y_{1\mathrm{st}}$ and $y_{2\mathrm{nd}}$, which are the partial matrices composed of $y_{l}$ for l=0,1,...,L−2 and l=1,1,...,L−1, respectively. Let $y_{▵}$ denote the difference between the two partial matrices; that is, $y_{▵}=y_{1\mathrm{st}}-y_{2\mathrm{nd}}$. In general, the clutters that do not move do not generate Doppler. This means that $y_{l}$ is the same, regardless of *l*, ignoring the effect due to the noise component. Therefore, in $y_{▵}$, only the signals corresponding to targets whose velocity is not zero remain. Then, the FFT operation is performed on the sampled beat signal, $y_{▵}$, in order to estimate the range of targets. For the convenience of notation, the lth ramp signal of $y_{▵}$ is denoted as $y_{l}\left[n\right]$. The kth FFT output of $y_{l}\left[n\right]$ is represented by $Y_{l}\left[k\right]$, and is calculated as

(18) $$\begin{array}{c} Y_{l}\left[k\right]=\sum_{n=0}^{N-1}y_{l}\left[n\right]\exp\left(-j2\pi nk/N\right)\;\;\mathrm{for}\;\;0\leq k\leq N-1, \end{array}$$

where *N* is the size of the FFT, which is a power of 2. The FFT output $Y_{l}\left[k\right]$ is expressed, in vector form, as

(19) $$\begin{array}{c} Y_{l,\mathrm{FFT}}=D{\tilde{y}}_{l}, \end{array}$$

where D is an N×N matrix for the discrete Fourier transform operation, which consists of *L* column vectors; that is, $D=[D_{0},D_{1},...,D_{L-1}]$, where $D_{u}$ is the uth column vector of D (i.e., $D_{u}={\left[1,\;\;\exp\left(-j2\pi u/N\right),\exp\left(-j4\pi 2u/N\right),...,\exp\left(-j2\pi u(N-1)/N\right)\right]}^{T}$ for 0≤u≤N−1). Furthermore, ${\tilde{y}}_{l}\in C^{N\times 1}$ is vector concatenating $y_{l}$ and $0_{N-N_{s}}$ for zero-padding (i.e., ${\tilde{y}}_{l}={\left[y_{l}^{T}0_{N-N_{s}}^{T}\right]}^{T}={\left[{\underbrace{y_{l}\left[0\right],\;y_{l}\left[1\right],\ldots ,y_{l}\left[N_{s}-1\right]}}_{Ns},\;{\underbrace{0,\;0,\;\ldots ,\;0}}_{N-Ns}\right]}^{T}$, where $0_{N-N_{s}}\in R^{(N-N_{s})\times 1}$ is a zero vector). By peak detection of the magnitude of the FFT outputs, the estimated beat frequency, ${\hat{f}}_{b,m}$, can be obtained. By substituting ${\hat{f}}_{b,m}$ into Equation (15), the estimated range, ${\hat{d}}_{m}^{\mathrm{FFT}}$, is obtained.

### 3.2. Fine Range Estimation Using MUSIC

As shown in Figure 2, after coarse range estimation using the FFT, fine range estimation is performed by the MUSIC algorithm, which can achieve a higher resolution, compared with the FFT. The MUSIC algorithm achieves significantly higher resolution performance than the FFT by using the orthogonality between the noise and signal subspaces. The matrix form of the beat signal is denoted by: Let $y\in C^{N_{s}\times L}$ denote the matrix form of the beat signal (i.e., $y=\left[y_{1},y_{2},...,y_{L}\right]$). Let R denote the correlation matrix of y, expressed as follows [18]:

(20) $$\begin{array}{c} \begin{array}{c} R=yy^{H}\\ \;\;\;=\left[y_{0},y_{1},...,y_{L-1}\right]{\left[y_{0},y_{1},...,y_{L-1}\right]}^{H}\\ \;\;\;=\left[\begin{array}{ccc} y_{0}\left[0\right] & \cdots & y_{L-1}\left[0\right]\\ y_{0}\left[1\right] & \cdots & y_{L-1}\left[1\right]\\ \vdots & \ddots & \vdots \\ y_{0}\left[N_{s}-1\right] & \cdots & y_{L-1}\left[N_{s}-1\right] \end{array}\right]\left[\begin{array}{ccc} y_{0}{\left[0\right]}^{*} & \cdots & y_{0}{\left[N_{s}-1\right]}^{*}\\ y_{1}{\left[0\right]}^{*} & \cdots & y_{1}{\left[N_{s}-1\right]}^{*}\\ \vdots & \ddots & \vdots \\ y_{L-1}{\left[0\right]}^{*} & \cdots & y_{L-1}{\left[N_{s}-1\right]}^{*} \end{array}\right]\\ \;\;\;=\left[\begin{array}{cccc} R_{0,0} & R_{0,1} & \cdots & R_{0,L-1}\\ R_{1,0} & R_{1,1} & \cdots & R_{1,L-1}\\ \vdots & \vdots & \ddots & \vdots \\ R_{N_{s}-1,0} & R_{N_{s}-1,1} & \cdots & R_{N_{s}-1,L-1} \end{array}\right], \end{array} \end{array}$$

where ${\left(\cdot \right)}^{H}$ is the Hermitian operator and $R_{i,j}$ is the element at the ith row and jth column of R. By performing SVD on R, the signal and noise subspaces can be separated as [18]

(21) $$\begin{array}{ccc} R & = & U\Sigma U^{H}\\ & = & \underset{\mathrm{subspace}\;\mathrm{of}\;\mathrm{signal}}{\underbrace{U_{M}\Sigma_{M}U_{M}^{H}}}+\underset{\mathrm{subspace}\;\mathrm{of}\;\mathrm{noise}}{\underbrace{\sigma_{n}^{2}U_{-M}}}, \end{array}$$

where $U_{M}$ is the subspace of the signal (i.e., $U_{M}=\left[u_{1},u_{2},...,u_{M}\right]$); $U_{-M}$ corresponds to the subspace of the AWGN component (i.e., $U_{-M}=\left[u_{M+1},u_{M+2},...,u_{N_{s}}\right]$); and Σ is a diagonal matrix based on $N_{s}$ eigenvalues (i.e., $\Sigma =diag(\lambda_{1},\lambda_{2},...,\lambda_{N_{s}})$, where $\lambda_{p}$ is the pth eigenvalue of R and diag(·) is the diagonal matrix operator). The ith eigenvalue, $\lambda_{i}$, is given as

(22) $$\begin{array}{c} \lambda_{i}=\left\{\begin{array}{cc} \rho_{i}+\sigma_{n}^{2} & 1\leq i\leq M\\ \sigma_{n}^{2} & M+1\leq i\leq N_{s}, \end{array}\right. \end{array}$$

where $\rho_{i}$ corresponds to the ith eigenvalue of the considered signal part and $\sigma_{n}^{2}$ corresponds to the noise variance. The region of the beat frequency $f_{b,m}\in \left[f_{b,m}^{\left(\min\right)},f_{b,m}^{\left(\max\right)}\right]$ is divided into a grid of $N_{B}$ values (i.e., $d\in [f_{b,m}^{\left(0\right)},f_{b,m}^{\left(1\right)},...,f_{b,m}^{\left(N_{B}-1\right)}]$), where $f_{b,m}^{\left(\min\right)}$ and $f_{b,m}^{\left(\max\right)}$ are the considered minimum and maximum values of the beat frequency, respectively. Hence, the steering vector is denoted by $h(f_{b,m})$ and expressed as $h\left(f_{b}\right)={\left[\eta_{1}\left(f_{b,m}\right),\eta_{2}\left(f_{b,m}\right),...,\eta_{M}\left(f_{b,m}\right)\right]}^{T}$, where $\eta_{m}\left(f_{b,m}\right)=\exp\left(-j2\pi f_{b,m}nt_{s}\right)$ for 1≤m≤M. We employ the orthogonal property between the steering vector $h(f_{b,m})$ and the subspace of the noise term $U_{-M}$, as follows:

(23) $$\begin{array}{c} h^{H}\left(f_{b,m}\right)U_{-M}U_{-M}^{H}h\left(f_{b,m}\right)=0. \end{array}$$

Therefore, using Equation (23), the pseudo-spectrum of the MUSIC algorithm, $P_{\mathrm{MUSIC}}$, is calculated as

(24) $$\begin{array}{c} P_{\mathrm{MUSIC}}=\frac{1}{h^{H}\left(f_{b,m}\right)U_{-M}U_{-M}^{H}h\left(f_{b,m}\right)}. \end{array}$$

By using Equation (15) and the estimated beat frequency with high resolution in Equation (24), close targets that could not be distinguished in the FFT-based estimation can be successfully distinguished.

In this procedure, the proposed algorithm performs the MUSIC algorithm for only a part of $y_{l}$, instead of performing the MUSIC algorithm for all samples of $y_{l}$. As shown in Equation (7), the sampling frequency, $f_{s}$, is determined by $d_{\max}$. However, as this is based on the worst case (i.e., $d_{\max}$), $f_{s}$ can be reduced if the target is not $d_{\max}$. The proposed algorithm employs the reduced sampling frequency $f_{s}^{\prime }$ based on ${\hat{d}}_{m}^{\mathrm{FFT}}$, instead of $d_{\max}$. The reduced sampling frequency, $f_{s}^{\prime }$, is calculated as

(25) $$\begin{array}{c} f_{s}^{\prime }=\frac{4\mu {\hat{d}}_{m}^{\mathrm{FFT}}}{c}. \end{array}$$

As ${\hat{d}}_{m}^{\mathrm{FFT}}\leq d_{\max}$ in most cases, $f_{s}^{{}^{\prime }}$ is smaller than $f_{s}$. In Equation (17), by setting the ratio of ${\hat{d}}_{m}^{\mathrm{FFT}}$ and $f_{s}^{\prime }$ equal to the ratio of $d_{\max}$ and $f_{s}$, the proposed algorithm achieves the same $d_{}$ as the case based on $d_{\max}$ with $f_{s}$.

**Figure 3:**
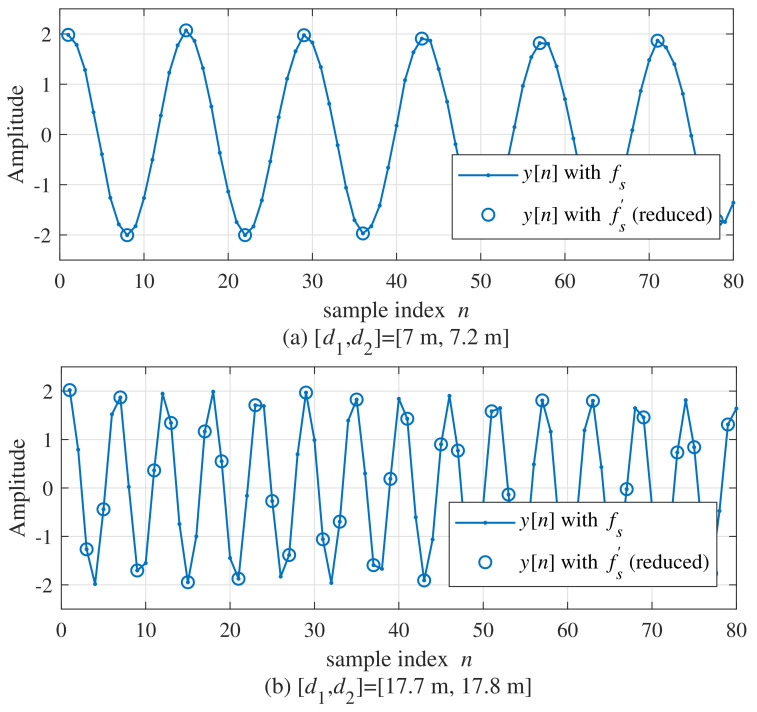
Comparison of waveforms between $y_{l}\left[n\right]$ with $f_{s}^{\prime }$ (reduced) and $f_{s}$ ((**a**)$\left[d_{1},\;d_{2}\right]=\left[7.0\;m,\;7.2\;m\right]$, (**b**)$\left[d_{1},\;d_{2}\right]=\left[17.7\;m,\;17.8\;m\right]$).

Figure 3 compares the waveforms of $y_{l}\left[n\right]$ between $f_{s}^{\prime }$ (reduced) and $f_{s}$. The ranges of the two targets are 7 and 7.2 m in Figure 3a and 17.7 and 17.8 m in Figure 3b, respectively. In this simulation, the considered maximum range $d_{\max}$ was set to 50 m and the range estimated by FFT ${\hat{d}}_{\mathrm{FFT}}$ was 7.1 m in Figure 3a. Hence, $f_{s}^{\prime }$ can be set to be about to 2.82 times lower than $f_{s}$. In the case of Figure 3b, as FFT ${\hat{d}}_{\mathrm{FFT}}$ was 17.75 m, $f_{s}^{\prime }$ can be set to about 1.94 times lower than $f_{s}$.

**Figure 4:**
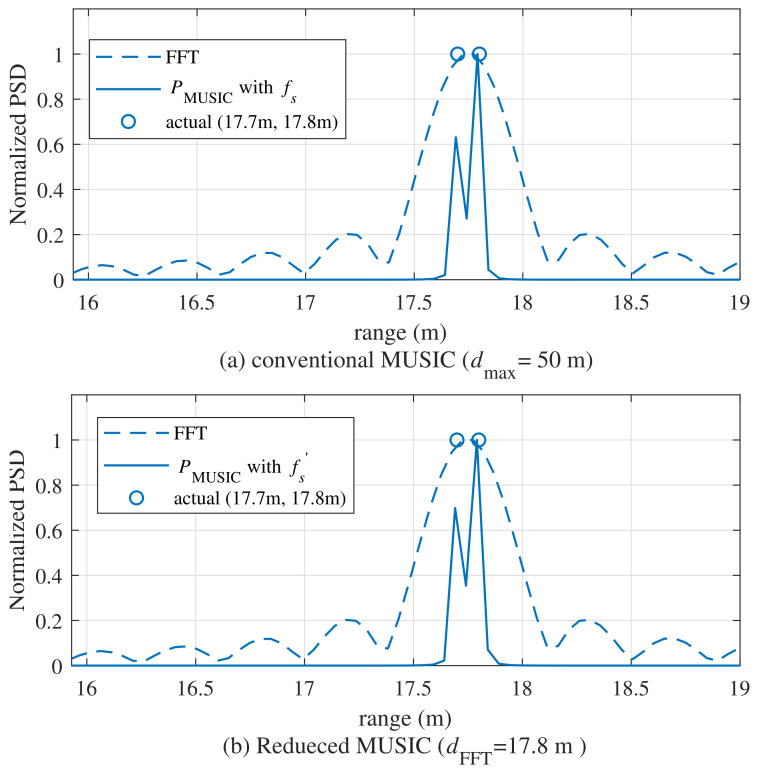
Comparison of $P_{\mathrm{MUSIC}}$ between the conventional MUSIC algorithm and the reduced-sample MUSIC algorithm((**a**)conventional MUSIC algorithm, (**b**) reduced MUSIC algorithm).

Figure 4 compares the range estimation results by $P_{\mathrm{MUSIC}}$ between the conventional MUSIC algorithm and the proposed algorithm with a reduced sample, where $\left[d_{1},\;d_{2}\right]=\left[17.7\;m,\;17.8\;m\right]$ and $d_{\mathrm{FFT}}=17.75\;m$. In Figure 4, as can be observed from the power spectral density (PSD) result using the FFT, they were estimated as one target, even though there were two targets. Compared with the FFT, the conventional MUSIC algorithm and the proposed MUSIC algorithm could estimate the two adjacent targets. In Figure 4b, the proposed MUSIC algorithm achieved similar estimation performance as the conventional MUSIC algorithm, despite using a reduced number of samples.

## 4. Performance Evaluation

### 4.1. Simulation Results

Here, we discuss the results of our simulations, in order to verify the improvement in the performance provided by the proposed super-resolution algorithm. For the simulations, the parameters $f_{0}$ and the maximum distance $d_{\max}$ were set to 24 GHz and 50 m, respectively. The number of targets was set to 2 (i.e., M=2) and the ranges of the two targets of each target, $d_{1}$ and $d_{2}$, were selected to be independent and uniformly distributed between 1 and $d_{\max}$. For the initial estimate, we performed 1024-point FFT. To generate various sample sizes, the bandwidth *B* was set to 1.54 GHz, 768 MHz, and 384 MHz (leading to $N_{s}=\left[1024,512,256\right]$). To calculate the RMSE, we performed $10^{4}$ simulations. As a measure to observe the performance difference between the conventional high-complexity algorithm and the proposed low-complexity algorithm, we calculated the root mean square error (RMSE) of the estimation of the range. The RMSE was calculated as $\mathrm{RMSE}=\sqrt{\frac{1}{M\times 10^{4}}\sum_{i=1}^{10^{4}}\sum_{m=1}^{M}{\left(d_{m}-{\hat{d}}_{m}\right)}^{2}}$.

**Figure 5:**
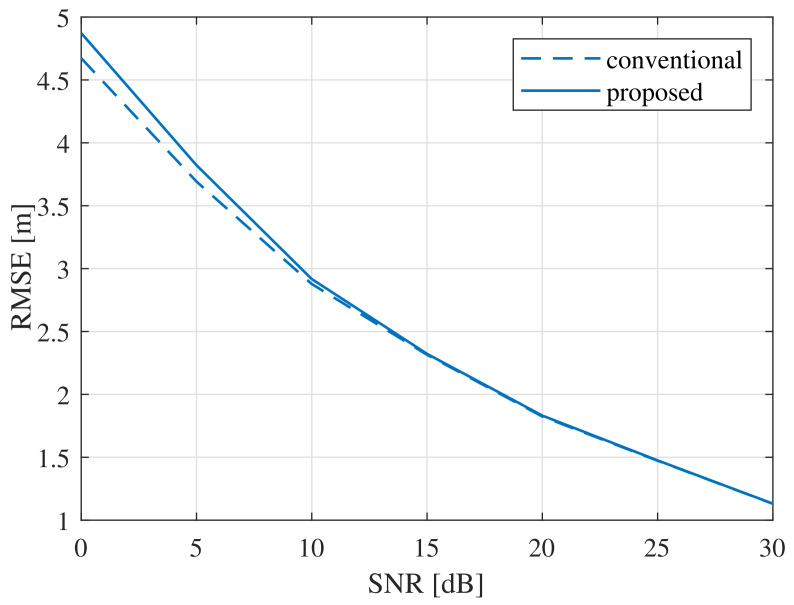
RMSE comparison for various sample sizes: $N_{s}=\left[1024,512,256\right]$.

Figure 5 shows the RMSE of the range estimations by the conventional and proposed MUSIC algorithms. In the low-SNR region (i.e., SNR = 0 dB), the RMSE of the proposed algorithm was about 4.5% higher, compared with that of the conventional MUSIC algorithm. However, the RMSE results of the two algorithms were almost the same when SNR ≥ 0 dB, despite the significantly lower computational complexity of the proposed MUSIC algorithm.

### 4.2. Complexity Comparison

In this section, the computational complexity of the proposed and conventional MUSIC algorithms was analyzed and compared. To measure the complexity of each algorithm, we compared the required number of multiplications for the generation of noise subspace and the SVD operation [43]. Let $C_{\mathrm{conventional}}$ and $C_{\mathrm{proposed}}$ denote the required number of multiplications in the conventional MUSIC algorithm and the proposed MUSIC algorithm, respectively. The case of the conventional algorithm, $C_{\mathrm{conventional}}$, was calculated as follows:

(26) $$\begin{array}{c} C_{\mathrm{conventional}}=\frac{16}{5}N_{s}^{3}+\frac{1}{2}N_{s}\left(N_{s}-M\right)\left(N_{s}+1\right). \end{array}$$

For the proposed MUSIC algorithm, the number of samples decreases with the initial estimated range $\hat{d}$; however, for the initial range estimation, the FFT operation is additionally required. Hence, $C_{\mathrm{proposed}}$ was calculated as

(27) $$\begin{array}{c} C_{\mathrm{proposed}}=\frac{N_{R}}{2}\log_{2}N_{R}+\frac{16}{5}{N_{s}^{{}^{\prime }}}^{3}+\frac{1}{2}N_{s}^{{}^{\prime }}\left(N_{s}^{{}^{\prime }}-M\right)\left(N_{s}^{{}^{\prime }}+1\right), \end{array}$$

where $N_{s}^{{}^{\prime }}$ is the reduced number of samples (i.e., $N_{s}^{{}^{\prime }}=\frac{\hat{d}}{d_{\max}}\times N_{s}$).

**Figure 6:**
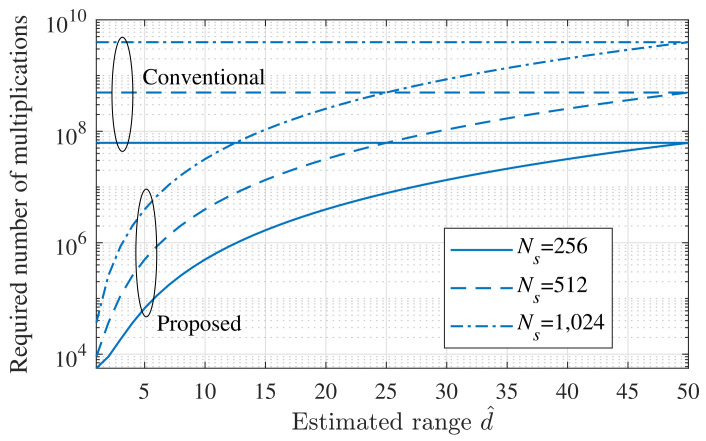
Required number of multiplications, according to the estimated range $\hat{d}$.

Figure 6 shows $C_{\mathrm{conventional}}$ and $C_{\mathrm{proposed}}$, with respect to the initial estimation of the range. In the worst case (i.e., $\hat{d}=d_{\max}$), the complexity of the two algorithms was almost the same. However, as $\hat{d}$ decreased, compared with $d_{\max}$, the complexity drastically decreased. In the case $\hat{d}=10\;m$, the proposed algorithm achieved a 99.17% complexity reduction, compared with the conventional algorithm. In addition, when $\hat{d}=20\;m$, the proposed algorithm achieved a 93.33% complexity reduction, compared with the conventional algorithm. In the general case, as *d* is smaller than $d_{\max}$, the complexity of the proposed algorithm was expected to be significantly lower, compared with the existing MUSIC algorithm. Assuming that the target distance was uniformly distributed between 1 m and $d_{\max}=50\;m$, the average range was $d_{\max}/2$. Assuming these conditions, the complexity of the proposed algorithm was reduced by about 88%, compared with the conventional algorithm.

## 5. Experiments

In this section, we describe the experiments we conducted using a real FMCW radar system, in order to verify the performance of the proposed MUSIC algorithm in a practical environment. First, we introduce the modules and equipment used in the experiment and their specifications; then, the measurement results are provided and analyzed.

### 5.1. Experimental Set-Up

**Figure 7:**
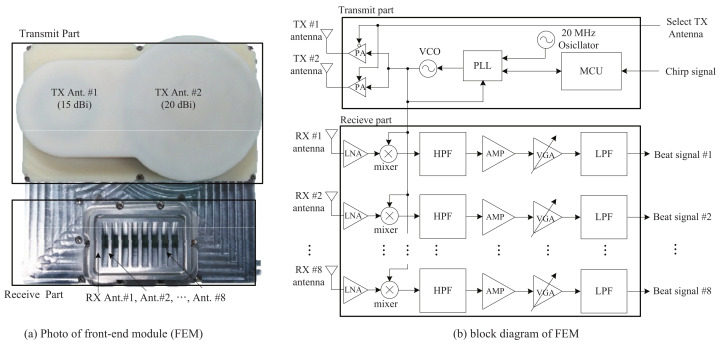
Photo and structure of the FEM.

Figure 7a,b show photos of the actual structure of the front-end module (FEM). As shown in Figure 7a,b, the FEM was composed of two parts, a TX part and an RX part. Two TX antennas were located on the top of FEM, with gains of 15 and 20 dBi. The azimuth and elevation angles of the RX antennas were 99.6${}^{\circ }$ and 9.9${}^{\circ }$, respectively. A power amplifier (PA), voltage-controlled oscillator (VCO), phase-locked loop (PLL), oscillator at 20 MHz, and a micro-controller unit (MCU) were included in the TX part. The frequency synthesizer with the PLL was controlled by the MCU, and one of the two TX antennas was selected. The azimuth angles of the first and second TX antennas were 26${}^{\circ }$ and 12${}^{\circ }$, respectively. The RX part was located at the bottom of the FEM. There were 8 RX antennas, and the distance between two adjacent RX antennas was half a wavelength. In the RX part, low-noise amplifiers (LNAs) were included for noise reduction. In addition, a mixer to obtain the beat signals and two kinds of filters—that is, high-pass filters (HPFs) and low-pass filters (LPFs)—were included. The amplifier (AMP) was used to amplify the weak signal, and the variable-gain amplifier (VGA) was used to control the gain, according to the input. The RX signals from the RX antennas passed through the LNA and thus, the noise terms in the RX signals were reduced. Then, the output of the LNA was multiplied by the TX signal, synchronized by the PLL. The mixed signals were input to the (150 kHz) HPFs and then amplified by the AMPs. Finally, the outputs of the AMPs were passed through the (1.7 MHz) LPF.

**Figure 8:**
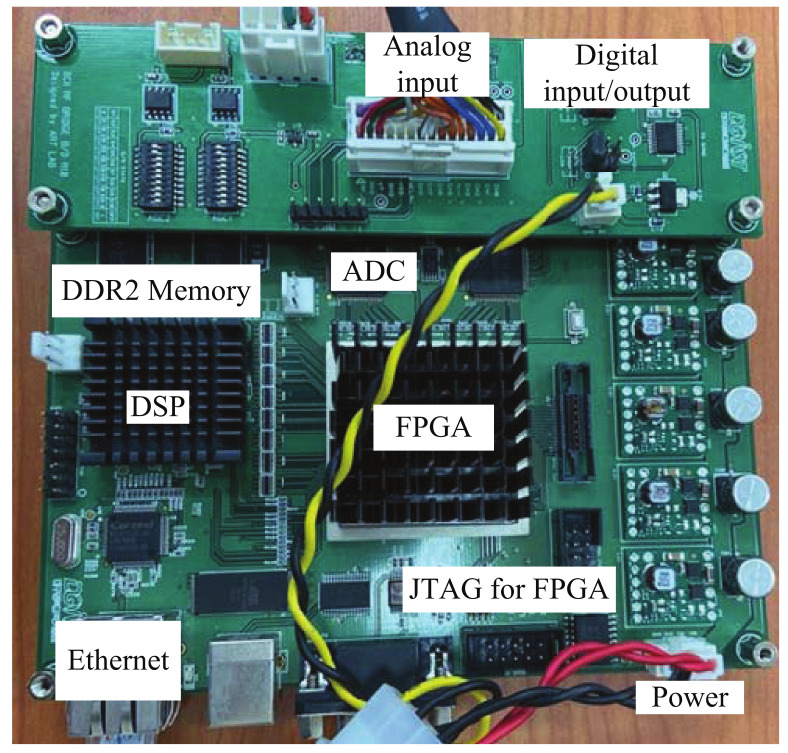
Photo of the BEM.

Figure 8 shows a photo of the back-end module (BEM). As shown in Figure 8, a field-programmable gate array and digital signal processing were included in the BEM. The analog signal from the FEM to the analog input was converted into a digital signal, with a 20 MHz sample rate, using an analog-to-digital converter. The converted signal was stored as data in two external memory banks with 512 Mbytes for DSP, called DDR2 SDRAMs. When the two DDR2 SDRAMs were filled with data, the stored data were moved to a personal computer (PC) using an ethernet cable.

**Figure 9:**
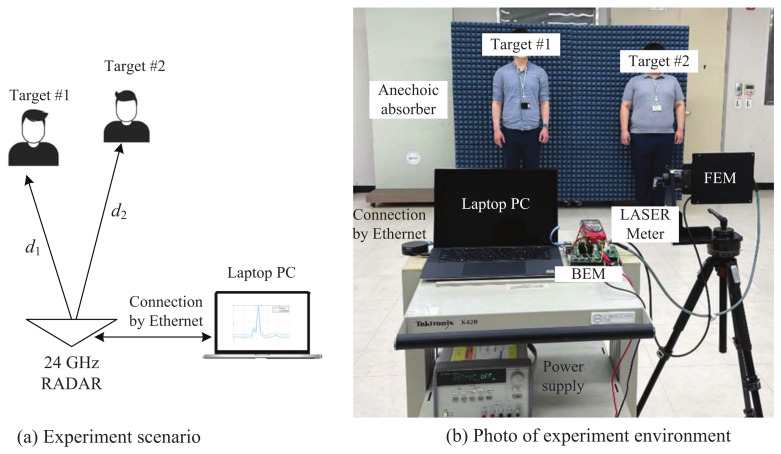
Scenario and environment used for the experiment ((**a**)experiment scenario (**b**) photograph of experiment environment).

Figure 9 shows the scenario and environment used for the experiment. As shown in Figure 9a,b, the targets were two people, who were $d_{1}$ and $d_{2}$ meters away from the radar, respectively. As mentioned above, the data of the sampled beat signal transformed by the ADC were transmitted to the PC by an ethernet cable. By employing software installed for the experiment, as shown in Figure 9b, we easily set and selected the parameters, such as the sampling rate, number of ramps, and so on. Then, the performance of each algorithm was verified by applying the proposed algorithm and the conventional algorithm to the same data.

### 5.2. Experimental Results

**Figure 10:**
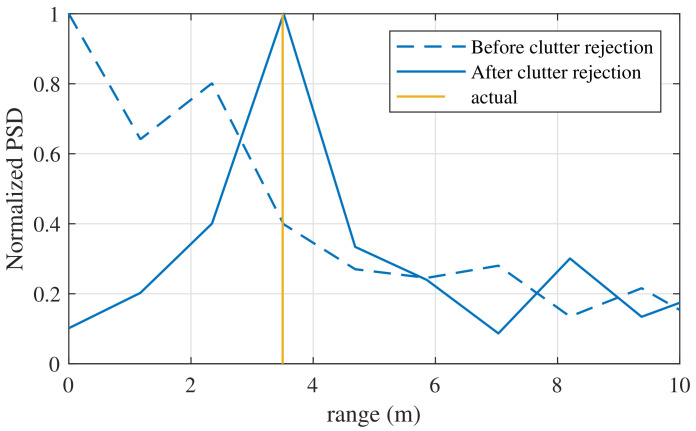
Comparison with the algorithm without clutter rejection.

Figure 10 shows the estimation results using FFT with the clutter rejection algorithm. The two targets were located at the same range of 3.5 m, and each angle was located at $\pm 20^{\circ }$. The two targets were not stationary objects but humans, and thus there is movement of the chest by breathing. Therefore, a Doppler change occurs due to the respiration of the targets and thus the clutters are easily canceled. In the case without the clutter rejection algorithm, we observed dominant peaks at 0 and 2.2 m, as shown in Figure 10. Hence, the ranges without clutter rejection were estimated as 1 m and 2.2 m. In contrast, according to the results of the clutter rejection algorithm, the dominant peaks at 0 and 2.2 m were removed. The result of the range estimation was almost identical to the actual range.

**Figure 11:**
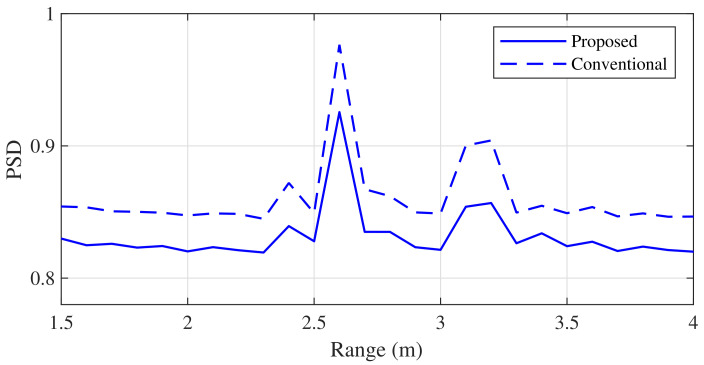
Comparison of the range estimation experiment results ($d_{1}=$2.7 m and $d_{2}=$3.2 m).

**Figure 12:**
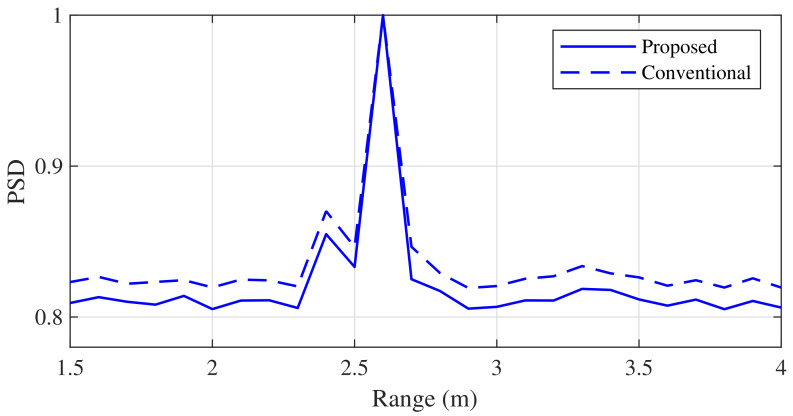
Comparison of the range estimation experiment results ($d_{1}=$2.4 and $d_{2}=$2.6 m).

Figure 11 and Figure 12 show the results of the range estimation experiment using the proposed and conventional MUSIC algorithms. The simple clutter rejection algorithm was applied to both algorithms. In Figure 11, the ranges of the two targets, $d_{1}$ and $d_{2}$, were 2.7 and 3.2 m, respectively. From these results, we observed that the two adjacent targets were properly distinguished by the proposed algorithm, despite its low complexity.

Figure 12 shows the experimental results when the distance between the two targets was closer than that in Figure 11 (i.e., $\left[d_{1},d_{2}\right]=\left[2.4\;m,2.6\;m\right]$). From these results, we found that two adjacent targets could be distinguished by the proposed algorithm, similarly to the conventional algorithm, even when the two targets were very close.

## 6. Conclusions

We constructed a low-complexity MUSIC algorithm based on the FFT-estimated beat frequency, and analyzed and compared the complexity of the proposed and conventional MUSIC algorithms. The proposed algorithm achieved a complexity reduction of 10 to 100 times, while producing similar performance to the conventional MUSIC algorithm. In addition, we experimentally confirmed the performance improvement provided by the proposed algorithm in a practical environment.

## Data Availability

Not applicable.
